# Association of higher plasma leptin levels with HOMA-IR index, high sensitivity C-reactive protein and glycolipid metabolism in patients with chronic schizophrenia: A multi-center cross-sectional study

**DOI:** 10.3389/fpsyt.2022.992988

**Published:** 2022-08-25

**Authors:** Zhiwei Liu, Yulong Zhang, Juan Wang, Lei Xia, Yating Yang, Liang Sun, Dapeng Zhang, Wenzheng Li, Xianhu Yao, Rongchun Yang, Yun Liu, Huanzhong Liu

**Affiliations:** ^1^Department of Psychiatry, The Third People's Hospital of Fuyang, Fuyang, China; ^2^Department of Psychiatry, Chaohu Hospital of Anhui Medical University, Chaohu, China; ^3^Anhui Psychiatric Center, Anhui Medical University, Chaohu, China; ^4^Department of Psychiatry, Chengdu Fourth People's Hospital, Chengdu, China; ^5^Department of Psychiatry, Hefei Fourth People's Hospital, Hefei, China; ^6^Department of Psychiatry, Maanshan Fourth People's Hospital, Maanshan, China

**Keywords:** chronic schizophrenia, leptin, insulin resistance, HOMA-IR, high sensitivity C-reactive protein, glycolipid metabolism

## Abstract

**Background:**

Previous research has revealed that plasma leptin levels were closely related to glycolipid metabolism in schizophrenic patients. Insulin resistance (IR) and high sensitivity C-reactive protein (hs-CRP) were involved in glucolipid metabolism disorders. This study explored the correlation between plasma higher leptin levels, homeostasis model assessment of insulin resistance (HOMA-IR) index, hs-CRP and glycolipid metabolism in patients with chronic schizophrenia (CS).

**Methods:**

322 subjects were enrolled, and the psychopathological symptoms of each patient were assessed by a 30-item Positive and Negative Syndrome Scale (PANSS_−30_). Patients' plasma leptin levels were measured by enzyme-linked immunosorbent assay (ELISA). Fasting blood glucose (FBG) levels were determined by oxidase method. Insulin levels were tested by electrochemiluminescence, and hs-CRP levels were tested by immunoturbidimetry. *IBM SPSS 22.0* was used for data analysis.

**Results:**

Compared to the lower leptin group, patients in the higher leptin group had significantly higher body mass index (BMI), total cholesterol (TC), triglycerides (TG), low-density lipoprotein (LDL-C), insulin, HOMA-IR and hs-CRP levels; and lower negative factor scores, cognitive factor scores, and PANSS total scores (*P* < 0.05). Plasma leptin levels in CS patients were positively correlated with BMI, TC, TG, LDL-C, insulin, HOMA-IR and hs-CRP levels, and were negatively correlated with gender (male = 1, Female = 2), positive factor scores, negative factor scores, cognitive factor scores and PANSS total scores. Multiple linear regression analysis revealed that gender, BMI, positive factor scores, PANSS total scores, FBG, LDL-C, insulin, HOMA-IR and hs-CRP levels were independent influencing factors of leptin levels in CS patients (*P* < 0.05).

**Conclusion:**

Gender, BMI, positive factor scores, PANSS total scores, FBG, LDL-C, insulin, HOMA-IR and hs-CRP levels were independent influencing factors of plasma leptin levels in CS patients. Plasma leptin, HOMA-IR and hs-CRP levels should be measured regularly in CS patients to prevent or treat the disorders of glucose and lipid metabolism comorbidity with schizophrenia patients in clinical diagnosis and treatment.

## Introduction

Schizophrenia is a kind of severe mental illness in clinical practice, characterized by high disability, high relapse rate, and high social risk. A national survey study in China noted that the 12-months prevalence of schizophrenia in the Chinese population was 6‰ (1). Compared to ordinary people, schizophrenic patients live 10–25 years shorter (2), and the leading cause of death is an increased risk of cardiovascular accidents due to abnormal glucose and lipid metabolism (3). Leptin is a class of proteins (16 KD in length) encoded by obesity genes, synthesized and secreted mainly by adipocytes, with biological effects such as reducing appetite, promoting glucose utilization, increasing energy expenditure, and improving insulin sensitivity, also known as anti-obesity hormones (4). Previous studies have pointed out that plasma leptin levels were increased in schizophrenia patients (5) and that leptin was closely related to weight gain (6, 7), abnormal glucose metabolism and dyslipidemia (5) in patients.

Systemic or local insulin resistance (IR) occurred when the efficiency of insulin in promoting glucose uptake and utilization was decreasing (8). And IR is involved in the central part of metabolic disorders in the body, which can induce metabolic syndrome, diabetes, and so on (9). Currently, IR is evaluated by the homeostasis model assessment of insulin resistance (HOMA-IR). HOMA-IR can effectively assess the body's sensitivity or resistance to insulin and can reflect the disorders of glucolipid metabolism in the early stage (8, 10). Former studies had shown that the IR index was significantly increased in schizophrenic patients with medication or not (11, 12). A prospective study noted that compared to individuals without IR, people with IR had a 1.4-fold higher risk of cardiovascular events and a 1.5-fold higher risk of diabetes (13). On the other hand, inflammation is an essential factor in maintaining tissue homeostasis and metabolic disease processes (14), and hs-CRP was closely related to glucolipid metabolism disorders which might be used as a specific indicator of inflammation level in humans beings (15). Previous literature has shown that serum CRP levels were significantly increased in schizophrenia patients compared to the healthy population (11, 16) and could predict glucolipid metabolism disorders in patients (5).

Various works of literature have indicated that plasma leptin levels in schizophrenia patients were positively correlated with the HOMA-IR index and this relationship persisted after pharmacological treatment (16, 17). Secondly, Kelly et al. found that plasma higher leptin levels in schizophrenia patients were related to increased CRP levels (18). Another study noted that the risk of developing insulin resistance in schizophrenia was closely related to CRP levels (19). A systematic review showed that IR associated with metabolic disorders depends on the stimulatory activation of inflammatory factors of immune or repair systems, and those chronic inflammatory responses might be a key predictor in the development of IR (20). Furthermore, genome-wide research has indicated that the exact biological mechanisms of obesity and mental disorders were insulin resistance and pro-inflammatory cytokines, which were involved in developmental disorders of brain function (21). In conclusion, plasma leptin, insulin resistance and CRP levels in schizophrenic patients are closely related to metabolic disorders in schizophrenia patients. However, few studies investigated the interrelation between leptin levels and HOMA-IR index and hs-CRP in CS patients. Therefore, in this study, we divided plasma leptin concentrations into higher level and lower-level groups in CS patients. Using the HOMA-IR represented IR, measured plasma hs-CRP levels, and explored the interrelation between leptin levels and HOMA-IR, hs-CRP and metabolic indexes such as blood glucose and blood lipids. Meanwhile, the independent influencing factors of plasma leptin levels in CS patients were explored.

## Methods

### Subjects

Three hundred twenty-two subjects were collected from Chaohu Hospital of Anhui Medical University, Hefei Fourth People's Hospital and Ma 'anshan Fourth People's Hospital. The time range of enrollment was from May 2018 to December 2018. Please refer to our previous manuscripts for sample size calculation (22, 23). Inclusion criteria: (1) age ≥18 years; (2) the diagnosis of patients with schizophrenia according to the 5^th^ edition of the Diagnostic and Statistical Manual of Mental Disorders (DSM-V); (3) Course of disease ≥5 years (24). (4) The patients have not used hormones, antibiotics and other drugs recently. Exclusion criteria: (1) combined mental retardation or severe neurological diseases; (2) women in pregnancy or lactation; (3) patients with severe physical diseases, such as cardiovascular, hepatic, respiratory, or renal diseases.

The patients or their guardians were informed by a specialized research member of the specific procedure of the trial and the possible risks and benefits during trail process and agreed to be enrolled and signed a paper version of the project informed consent form. This study was approved by the Ethics Committee of Chaohu Hospital affiliated to Anhui Medical University (No. 201805-kyxm-03) and obtained registration number (No. ChiCTR1800017044) from the China Clinical Trials Registry.

### Cases collection

The systematic case survey forms were used to record general socio-demographic information (gender, age, education, etc.,) of each subject; clinical information such as the patient's past medical history, medications category and dosage were obtained from the hospital's electronic medical record system or the supervising physician. Any unknown cases were supplemented by interviews with the patients or their families. The DDD conversion method recommended by WHO was used to convert antipsychotic drug treatment doses to chlorpromazine doses equivalently (25). Because of the severe weight gain effect of clozapine or olanzapine during medication treatment, patients on clozapine or olanzapine treatment grouped separately, and those taking the rest of the drugs (aripiprazole, amisulpride, ziprasidone, risperidone, quetiapine etc.,) were classified as weak weight gain drug (WWGD) group (26). Patients were divided into a higher leptin level group (≥*P*_75_) and a lower leptin level group (<*P*_75_) based on the quartiles of their plasma leptin levels (7, 27).

### Biochemical assays

Ten milliliters of venous blood were collected on the second day after the patients were enrolled. The lipid indexes such as total cholesterol (TC), triglycerides (TG), high-density lipoprotein (HDL-C), low-density lipoprotein (LDL-C) and fasting blood glucose (FBG), insulin and glucagon levels were tested by the corresponding laboratory staff. Plasma leptin concentrations were measured by the enzyme-linked immunosorbent assay (ELISA) method (kit: Cusabio Biotechnology Company, Wuhan, China). The calculation formula of the homeostasis model assessment of insulin resistance (HOMA-IR) is: HOMA-IR = [fasting insulin (μIU/ml) × fasting blood-glucose (mmol/L)]/22.5 (28). The calculation formula of body mass index (BMI) is: BMI = weight (kg) / [height (m)]^2^. FBG was determined by oxidase method (kit: Meikang Biotechnology Co., Ltd. Ningbo, China), insulin levels by electrochemiluminescence method (kit: Mannheim Roche Diagnostics Company, Shanghai, China), and glucagon by radioimmunoassay method (kit: Beijing North Institute of Biotechnology Co., Ltd. Beijing China). Hs-CRP levels were obtained by immunoturbidimetric centralized detection (kit: Beijing Leadman Biochemical Co., LTD. Beijing, China).

### Scale evaluation

Using the 30-item Positive and Negative Syndrome Scale (PANSS_−30_) to assess the psychiatric symptoms of each patient. The five-factor model was used for the statistical scoring (29). The positive factor scores included items P1, P3, P5, and G9, negative factor scores included items N1, N2, N3, N4, N6, and G7, cognitive factor scores included items P2, N5, and G11, depressive factor scores included items G2, G3, and G6, excited factor scores included items P4, P7, G8, and G14, and total scores of PANSS were calculated respectively. The intraclass correlation coefficient (ICC) of the subscale scores and PANSS total scores was above 0.85.

### Main outcome mMeasures

Compare the general data of patients in the higher leptin level group and lower leptin level group, analyze the relationship among plasma leptin levels, HOMA-IR, hs-CRP, and glucolipid metabolic index, and analyze the independent influencing factors of higher plasma leptin level in CS patients.

### Statistical analysis

*IBM SPSS 22.0* statistical software was used for data processing. The measurement data conformed to normal distribution were represented as (*x* ± *s*); if not, they were expressed as Median *(P*_25_*, P*_75_*)*. Categorical variables were described in terms of percentages and using the chi-square test for comparison between two groups. The correlations between patients' plasma leptin, HOMA-IR, hs-CRP levels and glucolipid metabolic indexes were analyzed using Spearman's rank correlation. The independent influencing factors of plasma leptin levels were analyzed by multiple linear logistic regression using leptin levels as the dependent variable. *P* < 0.05 was set as the statistical test level.

## Results

### Comparison between higher and lower leptin levels groups

Compared to patients in the lower leptin levels (LLL) group, patients in the higher leptin levels (HLL) group had significantly higher BMI, TC, TG, LDL-C, insulin, HOMA-IR, and hs-CRP levels, and lower negative factor scores, cognitive factor scores, and PANSS total scores (*P* < 0.05). Further analysis indicated that plasma leptin levels were 2.82 (1.63, 4.23) ng/ml in female patients, which was significantly higher than 0.55 (0.33, 1.20) ng/ml in males (*P* < 0.01). See Table 1.

**Table 1:** Comparison of general data and biological indicators between the high leptin levels (HLL) group and the low leptin levels (LLL) group.

**Variable**	**HLL group** ***N* = 80**	**LLL group** ***N* = 242**	**t/Z/χ^2^**	***P***	**Effect size**
Age (Years)	44.92 ± 13.22	45.03 ± 11.25	0.071	0.943^a^	0.009
Gender					*NA*
Male (%)	15 (18.75)	142 (72.45)	75.203	**<0.001** ^**c**^	
Female (%)	49 (81.25)	54 (27.55)			
Education (Years)	8 (7, 11)	8 (5, 11)	−0.952	0.341^b^	−0.077
Age of onset (Years)	26.66 ± 8.53	24 (19, 30)	−0.918	0.359^b^	−0.113
Illness duration (Years)	18.00 ± 10.99	18 (10, 28)	−1.351	0.177^b^	0.135
BMI	26.73 ± 3.67	23.20 ± 3.48	−7.743	**<0.001** ^**a**^	−0.999
Antipsychotic category (%)					
Clozapine /olanzapine	42 (65.63)	123 (62.75)	0.049	0.826^c^	*NA*
WWGD group	22 (33.37)	73 (37.24)			
Hematological index					
FBG (*mmol/L*)	4.70 (4.30, 5.18)	4.80 (4.30, 5.43)	−1.271	0.204^b^	0.137
TC (*mmol/L*)	5.19 (4.24, 5.12)	4.49 (3.93, 5.15)	−4.161	**<0.001** ^**b**^	−0.457
TG (*mmol/L*)	2.58 ± 1.25	1.74 (1.28, 2.49)	−4.20	**<0.001** ^**b**^	−0.299
HDL-C (*mmol/L*)	1.06 ± 0.24	1.00 (0.87, 1.20)	−0.274	0.784^**b**^	−0.041
LDL-C (*mmol/L*)	2.58 ± 0.51	2.35 ± 0.65	−2.980	**0.003** ^**a**^	−0.384
Leptin (*ng/mL*)	4.22 (3.46, 5.36)	0.69 (0.37, 1.39)	−13.41	**<0.001** ^**b**^	−3.765
Insulin (*mU/L*)	12.59 (8.49, 16.91)	6.76 (4.24, 10.15)	−7.443	**<0.001** ^**b**^	−0.782
Glucagon (*ng/L*)	145.50 (102.23, 222.28)	132.5 (76.13, 200.45)	−1.622	0.105^**b**^	−0.010
HOMA-IR	3.10 ± 2.02	1.89 ± 2.84	−6.432	**<0.001** ^**b**^	−0.456
hs-CRP(*ng/L*)	185.53 ± 100.30	138.37 ± 98.95	−4.341	**<0.001** ^**b**^	−0.475
Type II diabetes mellitus(%)	7 (8.75)	31 (12.81)	0.952	0.329^c^	*NA*
PANSS					
Positive factor scores	9 (7, 13)	10 (7, 15)	−1.778	0.075^b^	0.265
Negative factor scores	15.46 ± 6.45	18.41 ± 7.08	3.302	**0.001** ^**a**^	0.426
Cognitive factor scores	8.36 ± 2.98	9.26 ± 2.91	2.379	**0.018** ^**a**^	0.307
Depressive factor scores	7.01 ± 3.00	6 (4, 9)	−0.439	0.660^b^	−0.083
Excited factor scores	6 (5, 9)	8 (5, 10)	−1.747	0.081^b^	0.238
PANSS total scores	72.81 ± 24.30	79.56 ± 23.90	2.179	**0.030** ^**a**^	0.281
Chlorpromazine equivalent (mg/d)	424.94 ± 226.31	410 (250, 620)	−0.938	0.348^b^	0.152

BMI, body mass index; FBG, Fasting blood-glucose; TC, total cholesterol; TG, triglyceride; HDL-C, high-density lipoprotein; LDL-C, low density lipoprotein; PANSS, Positive and Negative Syndrome Scale.

a two-sample t test,

b Mann-Whitney U test,

c Pearson chi-square test.

Bold value means P < 0.05.

### Correlation analysis of leptin levels and metabolic indexes

**Figure 1:**
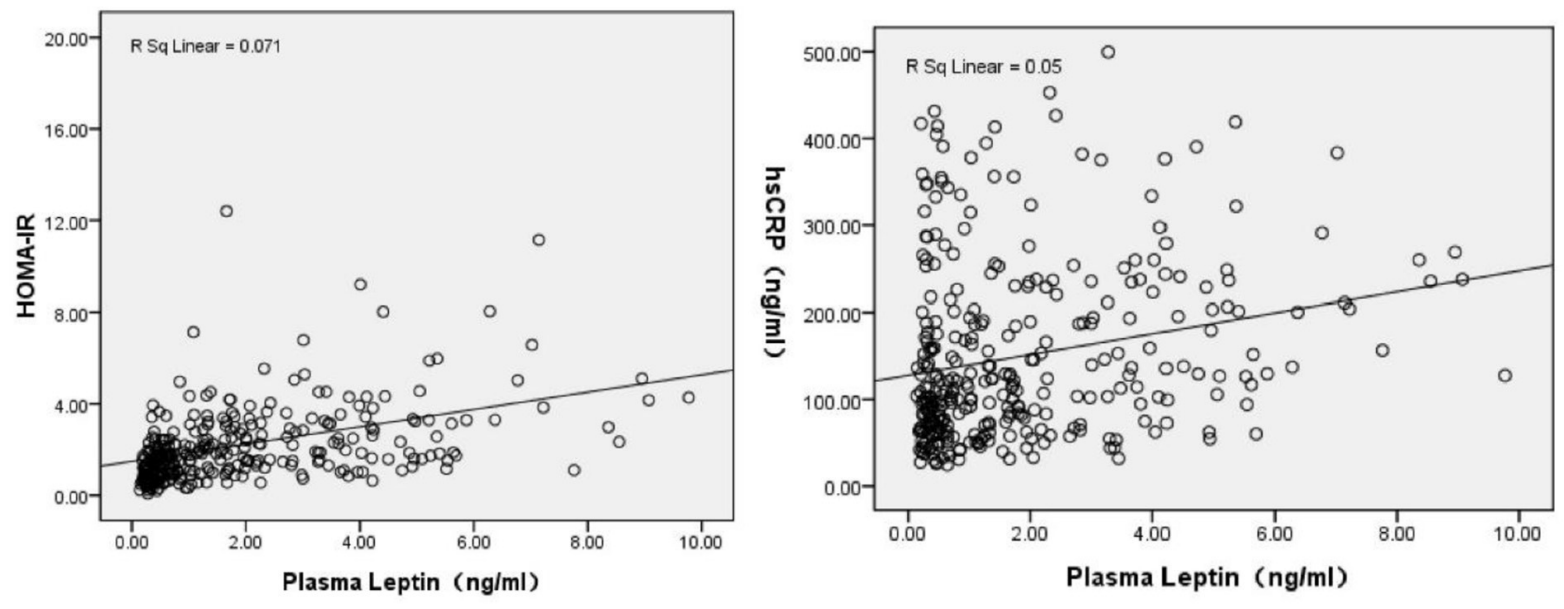
Correlation analysis revealed plasma leptin levels were positively associated with HOMA-IR (*r* = 0.543, *P* < 0.001), and hs-CRP levels (*r* = 0.250, *P* < 0.001).

Plasma leptin levels in CS patients were positively correlated with BMI (*r* = 0.572), TC (*r* = 0.268), TG (*r* = 0.313), LDL-C (*r* = 0.245), insulin (*r* = 0.595), HOMA-IR (*r* = 0.543) and hs-CRP levels (*r* = 0.250), and were negatively correlated with gender (male = 1, Female = 2) (*r* = −0.625), positive factor scores (*r* = −0.163), negative factor scores (*r* = −0.212), cognitive factor scores (*r* = −0.186) and PANSS total scores (*r* = −0.198), significantly. See Table 2. The correlation between plasma leptin levels and HOMA-IR and hs-CRP levels also shown in Figure 1.

**Table 2:** Correlation between leptin, HOMA-IR, hs-CRP levels and glucolipid metabolism index.

**Variables**	**Leptin**		**HOMA-IR**		**hs-CRP**	
	***r***	***P***	***r***	***P***	***r***	***P***
Age (Years)	0.018	0.741	−0.112	**0.047**	0.180	**0.001**
Gender	−0.625	**< 0.001**	−0.146	**< 0.001**	−0.061	0.275
Illness duration (Years)	−0.039	0.491	−0.095	0.090	0.098	0.080
BMI	0.572	**< 0.001**	0.468	**< 0.001**	0.224	**< 0.001**
FBG (*mmol/L*)	−0.025	0.653	0.378	**< 0.001**	0.114	**0.041**
TC(*mmol/L*)	0.268	**< 0.001**	0.232	**< 0.001**	0.095	0.090
TG(*mmol/L*)	0.313	**< 0.001**	0.424	**< 0.001**	0.121	**0.031**
HDL-C(*mmol/L*)	−0.043	0.437	−0.165	**0.003**	−0.181	**0.001**
LDL-C(*mmol/L*)	0.245	**< 0.001**	0.226	**< 0.001**	0.018	0.746
Insulin(*mU/L*)	0.595	**< 0.001**	0.944	**< 0.001**	−0.178	**0.001**
Glucagon(*ng/L*)	0.104	0.064	0.134	**0.017**	0.023	0.684
HOMA-IR	0.543	**< 0.001**	1	NA	0.199	**< 0.001**
hs-CRP(*ng/mL*)	0.250	**< 0.001**	0.199	**< 0.001**	1	NA
Positive factor scores	−0.163	**0.003**	−0.056	0.319	0.030	0.594
Negative factor scores	−0.212	**< 0.001**	−0.162	**0.004**	−0.090	0.107
Cognitive factor scores	−0.186	**0.001**	−0.126	**0.025**	−0.047	0.396
Excited factor scores	−0.164	**0.003**	−0.050	0.371	0.012	0.824
Depressive factor scores	−0.054	0.335	−0.033	0.554	0.044	0.428
PANSS total scores	−0.198	**< 0.001**	−0.123	**0.028**	−0.014	0.805

*Bold value means P < 0.05*.

### Correlation analysis of HOMA-IR with metabolic indexes

Plasma HOMA-IR levels in CS patients were positively correlated with BMI, FBG, TC, TG, LDL-C, insulin, glucagon, and hs-CRP levels, and negatively correlated with age, gender (male = 1, female = 2), HDL-C levels, negative factor scores, cognitive factor scores and PANSS total scores (*P* < 0.05). See Table 2.

### Correlation analysis of hs-CRP levels with metabolic indicators

Plasma hs-CRP levels in CS patients were positively correlated with age, BMI, FBG, TG levels and HOMA-IR, and negatively correlated with HDL-C and insulin levels (*P* < 0.05). See Table 2.

### Influencing factors of plasma leptin levels in CS patients by multiple linear regression analysis

We set plasma leptin levels as the dependent variable, and input general demographic data (gender, age, illness duration) and clinical variables (FBG, TC, TG, LDL-C, insulin, glucagon, HOMA-IR, hs-CRP, positive factor scores, negative factor scores, cognitive factor scores, excited factor scores, depressive factor scores, PANSS total scores) as independent variables, then results showed that gender (*B* = −1.847, *t* = −12.294, *P* < 0.001), BMI (*B* = 0.091, *t* = 4.184, *P* < 0.001), positive factor scores (*B* = −0.070, *t* = −2.733, *P* = 0.007), PANSS total scores (*B* = 0.024, *t* = 2.157, *P* = 0.032), FBG (*B* = 0.147, *t* = 2.114, *P* = 0.035), LDL-C (*B* = 0.322, *t* = 2.223, *P* = 0.027), insulin (*B* = 0.261, *t* = 8.015, *P* < 0.001), HOMA-IR (*B* = −0.661, *t* = −6.718, *P* < 0.001) and hs-CRP (*B* = 0.002, *t* = 2.790, *P* = 0.006) levels were independent influencing factors of leptin levels in CS patients (*F* = 24.803, *P* < 0.05). The R^2^ of the overall model was 0.612, and the adjusted R^2^ was 0.587. See Table 3.

**Table 3:** Influencing factors of plasma leptin levels by multiple linear regression analysis.

**Variables**	**Unstandardized coefficients**		***T***	***P***	**95% Confidence interval for B**	
	***B***	***Std. Error***			**Lower bound**	**Upper bound**
(Constant)	−1.847	0.845	−2.187	0.030	−3.510	−0.185
Gender	−1.871	0.152	−12.294	<**0.001**	−2.171	−1.572
Age (years)	0.010	0.009	1.138	0.256	−0.008	0.028
Illness duration (years)	−0.007	0.010	−0.757	0.450	−0.027	0.012
BMI	0.091	0.022	4.184	<**0.001**	0.048	0.134
FBG (*mmol/L*)	0.147	0.069	2.114	**0.035**	0.010	0.283
TC (*mmol/L*)	−0.034	0.068	−0.501	0.617	−0.168	0.100
TG (*mmol/L*)	−0.040	0.053	−0.756	0.450	−0.143	0.064
HDL-C (*mmol/L*)	−0.212	0.304	−0.698	0.486	−0.810	0.386
LDL-C (*mmol/L*)	0.322	0.145	2.223	**0.027**	0.037	0.606
Insulin (*mU/L*)	0.261	0.033	8.015	<**0.001**	0.197	0.325
Glucagon (*ng/L*)	0.000	0.000	−0.901	0.368	−0.001	0.000
HOMA-IR	−0.661	0.098	−6.718	<**0.001**	−0.854	−0.467
hs-CRP (ng/ml)	0.002	0.001	2.790	**0.006**	0.001	0.004
Positive factor scores	−0.070	0.026	−2.733	**0.007**	−0.120	−0.020
Negative factor scores	−0.026	0.020	−1.296	0.196	−0.066	0.014
Cognitive factor scores	−0.028	0.037	−0.757	0.450	−0.100	0.045
Excited factor scores	−0.046	0.033	−1.404	0.161	−0.111	0.019
Depressive factor scores	−0.010	0.036	−0.272	0.786	−0.082	0.062
PANSS total scores	0.024	0.011	2.157	**0.032**	0.002	0.047

*Bold value means P < 0.05*.

## Discussion

This study pointed out that BMI was significantly higher in the HLL group, and was closely related to plasma leptin, HOMA-IR and hs-CRP levels. As we know, leptin can be involved in regulating the body's eating behavior, which in turn regulates the level of glucolipid metabolism (30). Previous literature have shown that increased leptin levels were significantly associated with BMI, body weight (7), increased waist circumference, and energy intake in psychosis (31, 32). Follow-up studies have also found that the clinical symptoms of schizophrenic patients improved after taking antipsychotic drugs, but BMI increased and was associated with increased leptin levels, significantly (33, 34). In this paper, we pointed out that plasma leptin levels, HOAM-IR, and hs-CRP levels were positively correlated with BMI of CS patients, suggesting that inflammation level and endocrine function in patients might be closely related to weight gain. Accumulating evidence demonstrates that schizophrenia is characterized by systemic inflammation and disturbances in metabolism (35). Furthermore, insulin resistance, neurohormone activation and chronic inflammation were the main factors that induce metabolic disorders (36).

Our research found plasma leptin levels were positively correlated with TC, TG and LDL-C levels in CS patients. Previous literature in schizophrenia shown that leptin levels were positively associated with TC, TG (37) and percentage of body fat (32) and negatively related to HDL-C (31). However, our study did not reveal a correlation between leptin and HDL-C levels. Similarly, the reduction in lipid levels in schizophrenic patients after weight loss was significantly correlated with the decrease in leptin concentration (38). Leptin was at a high level in the early stage of mental illness or in the late stage of treatment, and was closely related to the high incidence of abnormal blood lipid metabolism (34).

This study indicated that plasma leptin levels were higher in female patients [2.82 (1.63, 4.23) ng/ml] than in male patients [0.55 (0.33, 1.20) ng/ml]. That gender could be an independent influencing factor on plasma leptin levels. A previous study showed that obesity rates of female CS patients were higher than male, significantly (39). And the correlation between leptin levels and weight gain was more significant in female patients (6). Consistent with this article, Baptista et al. also revealed the difference between female and male, and it was not related to the type of medication taken (16). The current studies confirmed that higher leptin levels in female patients were associated with their specific dietary habits (e.g., excessive sugar intake) (6, 31). Moreover, the proportion of body fat in women is higher, and subcutaneous fat is the main fat, while visceral fat was the main fat in men. Subcutaneous fat produced more leptin than visceral fat, and as a result, men had lower plasma leptin levels than women (40). Another reason was that female estradiol secretion promoted leptin secretion from adipose tissue, which could not occur in men (41).

A meta-analysis showed that psychiatric patients routinely taking antipsychotic drugs had varying degrees of elevated plasma leptin levels (42). Another study (43) pointed out that leptin levels elevated in schizophrenic patients treated with olanzapine or clozapine than in those taking haloperidol or risperidone; and the increase in leptin levels was particularly related to higher BMI (44). Stip et al. (45) analyzed this by suggesting that patients with routinely treated schizophrenia had a significantly higher sensitivity to appetitive stimuli in the insular cortex, amygdala, and cerebellum, which in turn caused increased eating behavior (46). Although no differences were found between drug types and drug doses in patients in different leptin level groups in our study, chlorpromazine equivalents were slightly higher in patients in the higher leptin level group [424.94 ± 226.31 mg/d vs. 410 (250, 620) mg/d].

Although Current evidence demonstrated that high levels of leptin were an indirect consequence of weight gain during pharmacotherapy (7, 47), individualized dietary patterns should be developed in clinical treatment and medications should be selected that have less impact on patients' metabolic indicators.

Previous literature indicated that insulin levels were increased in schizophrenic patients compared to the healthy population (48, 49) and were positively related to leptin levels in the early course of the illness (31). During the treatment of schizophrenia, leptin levels and insulin levels were also positively correlated (37, 50). In addition, plasma leptin levels in schizophrenia patients were negatively correlated with positive symptoms (51), negative symptoms, depressive factor scores (52), general psychopathology and PANSS total scores (53), which were generally consistent with the our findings. However, it also indicated that patients' leptin levels were positively correlated with their positive symptoms, general psychopathology symptoms and PANSS total scores (54, 55). Thus, plasma leptin levels in schizophrenia patients may play an important mediating role between antipsychotic-induced disorders of glucolipid metabolism and clinical symptom remission (56).

Our study noted that there were positive correlations between plasma leptin, HOMA-IR and hs-CRP levels. And HOMA-IR and hs-CRP levels were independent influencing factors on higher plasma leptin levels. In addition, plasma HOMA-IR levels and hs-CRP levels were closely related to lipid metabolism index. Various previous studies could support the results of our research. Previous research revealed that plasma leptin was positively correlated with CRP levels in psychiatric patients, and they could be significant predictors of metabolic syndrome (18). A clinical trial found that HOMA-IR was positively correlated with TG in conventionally treated schizophrenic patients compared to healthy controls (37). Furthermore, Baptista et al. showed a positive correlation between plasma leptin levels and IR and CRP levels in CS patients (16, 17). In addition, compared to healthy people, the HOMA-IR and hs-CRP levels were significantly increased in schizophrenic patients, and they were positively correlated (11). Moreover, the higher risk of comorbid insulin resistance in schizophrenia was associated with higher levels of CRP, suggesting that prevention or treatment of inflammation may be an advantageous option for preventing or treating comorbid IR in schizophrenia (19). Meta-analyses also indicated that IR associated with metabolic disorders required activation of the chronic inflammatory or psychological stress axis (20). Related genomic studies have pointed out that obesity-related metabolic disorders shared the same biological mechanisms as mental disorders, such as insulin resistance and pro-inflammatory cytokines, which might lead to developmental disorders of brain function and accelerate or predict the process of physiological aging of the organism (21). In summary, there may be a link between the glucolipid metabolism disorders in schizophrenic patients and all three of higher levels of leptin, HOMA-IR, and hs-CRP. In the whole course of illness, the abnormal lifestyle or long-term medication history of patients might lead to metabolic disorders, and the occurrence of this condition was closely related to the high levels of leptin, insulin resistance and hs-CRP. With the recovery of mental illness and the improvement of metabolic indicators, the levels of leptin, insulin resistance and hs-CRP in the body decreased accordingly.

There are several limitations of the present research. Firstly, this is a cross-sectional study, and the causal relationship between plasma leptin levels and weight change, glucose, dyslipidemia, HOMA-IR and hs-CRP levels could not be clarified. Secondly, the subjects included in this study were chronic schizophrenic patients, and the number of relapses and the long duration of medication led to more possible related confounding factors. Thirdly, this study did not investigate the matched healthy population plasma leptin levels, which could not show the difference between the two populations. Fourth, the differences in the inclusion subjects and blood index testing methods of our study have limited the cross-sectional and longitudinal comparisons with previous studies. Further rigorously designed multi-node follow-up studies are needed to investigate the exact relationship between the change of plasma leptin levels and abnormalities in HOMA-IR, hs-CRP, glucolipid metabolic indexes and psychopathological symptom of CS patients.

## Conclusion

Plasma leptin levels in CS patients were positively correlated with BMI, TC, TG, LDL-C, insulin, HOMA-IR, and hs-CRP levels, and were negatively correlated with gender (male = 1, Female = 2), positive factor scores, negative factor scores, cognitive factor scores and PANSS total scores. And gender, BMI, positive factor scores, PANSS total scores, FBG, LDL-C, insulin, HOMA-IR and hs-CRP levels were independent influencing factors of leptin levels in CS patients. Patients with CS should have their plasma leptin, HOMA-IR and hs-CRP levels regularly tested to prevent or treat disorders of glucolipid metabolism in clinical practice in CS patients. Furthermore, our findings support strong correlations between inflammation and high levels of leptin and IR in schizophrenic patients, which may partially explain the higher risk of metabolic disorders in CS patients comorbid with IR, and reducing chronic inflammation in the body in clinical practice may be a therapeutic target to prevent or treat comorbid metabolic disorders in schizophrenia.

## Data availability statement

The data analyzed in this study is subject to the following licenses/restrictions. The corresponding authors had full access to all the data collected in the survey. Only by receiving permission from the relevant organization(s) shall the data be shared with other researchers. Requests to access these datasets should be directed to HL, huanzhongliu@ahmu.edu.cn.

## Ethics statement

The studies involving human participants were reviewed and approved by the Biomedical Ethics Committee of Chaohu Hospital of Anhui Medical University (Grant no. 201805-kyxm-03). The patients/participants provided their written informed consent to participate in this study.

## Author contributions

HL originally designed the study, and have been responsible for obtaining funding and conceived the idea for this manuscript, supervised and checked the analysis, and the final draft. ZL, YZ, and JW wrote the first draft. ZL, LX, YL, LS, and DZ undertook data cleaning, checking and coding, and did the analysis with the help from XY and RY. ZL, YZ, JW, WL, XY, and RY participated in data collecting and analyzing. All authors contributed to this study, read the manuscript and approved the final manuscript. All authors contributed to the interpretation of data and the approval of the final report.

## Funding

This study was funded by grants from the National Natural Science Foundation of China (81801341), the Key Research and Development Projects in Anhui Province (1804h08020263), and the Scientific Research Foundation of the Institute for Translational Medicine (2017zhyx17).

## Conflict of interest

The authors declare that the research was conducted in the absence of any commercial or financial relationships that could be construed as a potential conflict of interest.

## Publisher's note

All claims expressed in this article are solely those of the authors and do not necessarily represent those of their affiliated organizations, or those of the publisher, the editors and the reviewers. Any product that may be evaluated in this article, or claim that may be made by its manufacturer, is not guaranteed or endorsed by the publisher.
